# A Recombinant MVA-Based RSV Vaccine Induces T-Cell and Antibody Responses That Cooperate in the Protection Against RSV Infection

**DOI:** 10.3389/fimmu.2022.841471

**Published:** 2022-06-14

**Authors:** Kathrin Endt, Yvonne Wollmann, Jana Haug, Constanze Bernig, Markus Feigl, Alexander Heiseke, Markus Kalla, Hubertus Hochrein, Mark Suter, Paul Chaplin, Ariane Volkmann

**Affiliations:** ^1^ Bavarian Nordic GmbH, Martinsried, Germany; ^2^ University of Zürich, Dekanat Vetsuisse-Fakultät Immunology, Zurich, Switzerland

**Keywords:** MVA, vaccines, RSV, protection, mode of action

## Abstract

Respiratory syncytial virus (RSV) causes a respiratory disease with a potentially fatal outcome especially in infants and elderly individuals. Several vaccines failed in pivotal clinical trials, and to date, no vaccine against RSV has been licensed. We have developed an RSV vaccine based on the recombinant Modified Vaccinia Virus Ankara-BN^®^ (MVA-RSV), containing five RSV-specific antigens that induced antibody and T-cell responses, which is currently tested in clinical trials. Here, the immunological mechanisms of protection were evaluated to determine viral loads in lungs upon vaccination of mice with MVA-RSV followed by intranasal RSV challenge. Depletion of CD4 or CD8 T cells, serum transfer, and the use of genetically engineered mice lacking the ability to generate either RSV-specific antibodies (T11µMT), the IgA isotype (IgA knockout), or CD8 T cells (β2M knockout) revealed that complete protection from RSV challenge is dependent on CD4 and CD8 T cells as well as antibodies, including IgA. Thus, MVA-RSV vaccination optimally protects against RSV infection by employing multiple arms of the adaptive immune system.

## Introduction

Respiratory syncytial virus (RSV) is a leading cause of respiratory illness in children and elderly individuals. The immunological determinants that contribute to complete protection against RSV are not fully understood. Investigations on potential immune correlates of protection against RSV infection have demonstrated that antibody responses established after natural RSV infections are poorly protective against reinfection and high levels of serum antibodies do not always correlate with protection (1–3). On the other hand, protection from RSV reinfection in mice is only partial in the absence of RSV-specific antibodies (4, 5), and RSV neutralizing antibody titers correlate with protection in both young and aged BALB/c mice vaccinated with RSV F protein (6). Apart from antibody responses, the cellular immune response is believed to play an essential role for RSV clearance from the lungs. Earlier studies showed that adoptive transfer of memory T cells from previously primed mice could clear persistent infection in athymic nude or irradiated mice and that regulatory T-cells play an important role in viral clearance after an RSV infection (7–9). Furthermore, CD4 as well as CD8 T-cells are involved in the termination of RSV replication and both T-cell subsets play a role in shortening the duration of RSV shedding and generation of protective immunity (10, 11).

The novel RSV vaccine candidate (MVA-RSV) is based on the highly attenuated virus vector, Modified Vaccinia Ankara Bavarian Nordic (MVA-BN^®^; registered as IMVANEX^®^/IMVAMUNE^®^/JYNNEOS^®^), encoding the RSV fusion protein (F), glycoprotein (G), nucleoprotein (N), and transcription elongation factor (M2-1) derived from RSV subtype A, as well as another G of RSV subtype B. The various surface proteins are expected to induce a broad humoral and cell-mediated immunity against all RSV-specific antigens. The G protein of both subtypes has been included since it differs substantially between subtypes, and thus, this vaccine may provide broad protection against RSV (A and B) disease. Indeed, MVA-RSV has been proven as a safe vaccine with the potential to elicit both T-cell and antibody responses as demonstrated in a phase 1 and phase 2 clinical trial (12, 13). Moreover, MVA-RSV demonstrated a vaccine efficacy of 79.3% in preventing moderately symptomatic RSV infection in a recent human challenge trial (14).

In our studies, we used two mouse models to shed light on MVA-RSV-induced immune mechanisms of protection against RSV infection in more detail, since mice can easily be manipulated either by depletion of certain cell types or by using genetic knockout mice in the laboratory setting. BALB/c mice, which show an intermediate susceptibility to RSV, have been widely used to study vaccine immunity and efficacy against RSV (11, 15). To complement and confirm data in this well-established mouse model, we also utilized genetically modified mice on the C57BL/6 background. In both mouse strains, a prime-boost vaccination with MVA-RSV provided sterilizing immunity, indicated by the absence of infectious virus and the complete lack of viral transcripts 4 days after RSV challenge. This provided the basis to study the role of CD4 and CD8 T cells as well as antibodies for complete protection. In MVA-RSV-vaccinated BALB/c mice, CD4 or CD8 T cells were depleted, or serum from vaccinated mice was transferred to naïve mice upon RSV challenge. On the C57BL/6 background, CD8 T-cell-deficient mice (β2M -/-), antibody-deficient (T11µMT) mice, or IgA-deficient (IgA -/-) mice were employed for vaccination and RSV challenge, with the aim to define potential correlates of protection. Here, instead of a single correlate of protection, we show that the immune mechanisms required for RSV protection after vaccination with MVA-RSV involve all adaptive immune responses examined.

## Materials and Methods

### Animals

BALB/c (8 to 9 weeks of age) and C57BL/6 (13 weeks of age) female mice were purchased from Janvier Labs. IgA-deficient mice (IgA -/-) at the age of 12 to 24 weeks, CD8-deficient mice (β2M -/-) at the age of 8 weeks, and T11µMT transgenic mice (T11µMT) at the age of 20 to 24 weeks, all on a C57BL/6 background, were provided by the University of Zurich, Laboratory Animal Service Center. Mice were kept under specific-pathogen-free conditions in the animal facility of Bavarian Nordic. Mouse experiments were performed in compliance with the German Animal Welfare Law (Deutsches Tierschutzgesetz) and approved by the government of Upper Bavaria (approval no. 55.2-1-54-2532-112-2016).

### Vaccines and Viruses

MVA-RSV is based on MVA-BN^®^ (ECACC cat no. V00083008) as the backbone virus (16) and encodes the full-length version of the membrane-anchored fusion protein F (A long), the full-length version of the membrane-anchored glycoprotein G (A) and G (B), as well as the full-length version of the nucleoprotein N and transcription elongation factor M2-1 from the A2 strain. The latter (N and M2-1) are expressed as fusion protein. The RSV genes were inserted in specific Intergenic Regions (IGR) of the MVA-BN genome. RSV-G (A) and RSV-G (B) were inserted in IGR 64/65 and RSV-N, RSV-M2-1, and RSV-F were inserted in IGR 148/149. Expression of transgenes was driven by synthetic or natural occurring pox virus promoters. No marker gene was present in the recombinant virus. The coding sequence of RSV-G (A) was based on the naturally occurring glycoprotein G sequence of the RSV-A2 strain whereas the coding sequence of RSV-G (B) was based on the naturally occurring glycoprotein G sequence of the RSV B strain. The DNA sequence was codon optimized in such way that the least possible sequence homology between both glycoprotein variants exists. Both inserted genes were synthesized by GeneArt with optimized codon usage and used for cloning into the recombination plasmid. Expression of RSV-G (A) was driven by the strong early and late promoter Pr7.5e/l, which naturally transactivates gene expression of the Vaccinia virus 7.5-kDa gene. Expression of RSV-G (B) was driven by the synthetic promoter PrS, which was designed from consensus sequences of early and late elements of Vaccinia virus promoters. The coding sequences for RSV-N and RSV-M2-1 were based on the naturally occurring sequences of the RSV A2 strain. Both genes were connected by a well-characterized 2A self-cleaving peptide sequence of the foot-and-mouth disease virus that allowed the expression of two separate native proteins under the control of a single promoter. The coding sequence of RSV-F was based on the RSV-A_long_ strain. The genes were synthesized by GeneArt with optimized codon usage. Expression of RSV-N and RSV-M2-1 was driven by the synthetic promoter PrLE1 designed by BN, which is a fusion of the late cowpox virus A type inclusion promoter with optimized early elements of the Pr7.5e/l promoter. Expression of RSV-F was driven by the synthetic promoter PrH5m, which was a modified version of the Vaccinia virus H5 gene promoter consisting of strong early and late elements.

MVA-RSV was generated by homologous recombination. For this purpose, primary chicken embryo fibroblast (CEF) cells were infected with MVA-BN and subsequently transfected with the appropriate recombination plasmids. During homologous recombination, the sequences within the plasmid homologous to the insertion sites of the MVA-BN genome recombine with their corresponding sequences within the viral genome and target the transgenes into the respective integration site (either IGR 64/65 or IGR 148/149) of MVA-BN. MVA-RSV was further propagated in CEF cells at serum-free conditions. After insertion of the antigens into the MVA-BN genome, genetically pure clones were isolated by repeated rounds of limiting dilution and plaque purification. A final clone was amplified, and a stock was prepared, which was extensively analyzed, including proof of (i) correct size, (ii) correct sequence of the inserts, (iii) absence of parental MVA-BN virus, (iv) and absence of extraneous microbes (sterility testing). In addition, the (v) infectious titer of the MVA-RSV virus stock was determined. Such virus stock was used as inoculum during production of research grade material for use in animal vaccine studies. Production was conducted in roller bottles seeded with primary CEF cells under serum-free conditions. Infected CEF lysates were sonicated, purified, and concentrated using a standardized two-step sucrose cushion centrifugation procedure. Vaccine infectious titer, sequence identity, and integrity were confirmed. Transgene expression was confirmed at various developmental steps by Western blot and flow cytometry, confirming extracellular expression of the RSV-derived genes F and G.

### Immunization and Challenge

Mice were administered intranasally (IN) with 100 µl of the MVA-RSV vaccine at 1 × 10^8^ TCID_50_ per dose (1 TCID_50_ corresponds to 1 infectious unit [Inf.U]) at Days 0 and 21. IN challenge was performed with 100 µl of RSV-A2 at 1 × 10^6^ pfu at Day 35 (9, 15). Control animals received TRIS-buffered saline, pH 7.7. For IN applications, mice were anesthetized with a mixture of Fentanyl, Midazolam, and Medetomidine and anesthesia was antagonized with a mixture of Naloxone, Flumazenil, and Atipamezole. After challenge, animals were monitored daily, and body weight was measured. Animals were sacrificed 4 days post challenge (peak of infection).

### T-Cell Depletion and Transfer of Donor Serum

CD4 or CD8 T-cell subsets were depleted in BALB/c mice by intraperitoneal injection of 250 µg of anti-CD4 (rat IgG2b, Clone GK1.5, Bio X Cell) or anti-CD8 (rat IgG2b, Clone 53-6.7, Bio X Cell) antibody 1 day before and 1 day after challenge. As control, an isotype-matched antibody (rat IgG2b, LTF-2, Bio X Cell) was used. For long-term depletion of CD4 T cells, injection was performed 2 days before prime vaccination and twice a week thereafter. At Day 39 (4 days post RSV-A2 challenge), the efficacy of these treatments was determined by FACS analysis of heparinized whole blood and splenocytes. Briefly, cells were treated with a purified anti-mouse CD16/CD32 monoclonal antibody (1:50, Fc Block™, BD) prior to staining with CD8 FITC (1:200; BioLegend) and CD4 brilliant violet 785 (1:200, BioLegend). For live versus dead cell discrimination, Zombie Aqua™ (1:200, BioLegend) was used. All cells were acquired using a digital flow cytometer (LSR II, BD Biosciences), and data were analyzed with FlowJo software (Tree Star).

Serum from MVA-RSV-vaccinated or mock-treated control mice was transferred by intraperitoneal (IP) injection of 1 ml of serum to non-vaccinated BALB/c mice 1 day before challenge.

### RSV Load in Lung

Mouse lung homogenates were generated and used for titration on Vero E6 cells (ATCC^®^ Number: CRL-1586™) in 48-well plates. In brief, 2‐fold serial dilutions of homogenized lung tissue supernatant (duplicate measurement) or RSV-A2 (1:500; positive control) were adsorbed onto a monolayer of Vero E6 cells. After adsorption, a solid overlay medium was applied to the cell monolayer to restrict virus growth to the originally infected foci of cells. Plates were incubated at 37°C and 5% CO_2_ for 6 days, following visualization of plaques after the addition of crystal violet solution. The plates were scanned and the numbers of plaques per well were counted automatically by a validated neural network. The average number of plaques adjusted by the respective dilution factor was then multiplied by 10 to obtain the titer of the solution in plaque-forming units [pfu]/ml.

Viral load was determined by reverse transcription quantitative polymerase chain reaction (RT-qPCR). RT reactions were performed using the High-Capacity RNA-to-cDNA Kit (Applied Biosystems). PCR specific for RSV-A2 L-gene was performed using Taqman Gene Expression Master Mix (Applied Biosystems), primer 1: GAACTCAGTGTAGGTAGAATGTTTGCA, primer 2: TTCAGCTATCATTTTCTCTGCCAAT, and probe: 6FAM-TTTGAACCTGTCTGAACATTCCCGGTT-MGB. Before the genomic copy numbers (gcs) per sample were calculated from the standard curve (pMISC202 plasmid vector containing a fragment of the RSV L-gene), all measured CT values were normalized to the18S housekeeping gene. Then, the normalized CT values were used to calculate the corresponding copy numbers for each sample from the standard curve. The detection limit of the assay was 15 copies, which was the corresponding copy number to the highest possible CT value of 40. RSV L-gene copies were measured by real-time PCR using the Applied Biosystems 7500 Real-Time PCR System and analyzed using the 7500 System Software.

### RSV-Specific Antibody Analysis

RSV-specific IgG levels in serum or bronchoalveolar (BAL) fluids were measured by a direct ELISA. 96-well ELISA plates were coated overnight with inactivated total RSV-A (RSV Grade 2 Antigen [Long Strain], Meridian Bioscience). Samples were titrated using serial dilutions starting at 1:100 for serum and 1:20 for BAL fluids. A sheep anti-mouse IgG-HRP (AbD Serotec) or a goat anti-mouse IgG-HRP (Abcam) was used as detection antibody for sera or BAL fluids, respectively. The antibody titers were calculated by a 4-parameter fit (Magellan Software). The average of the OD values of the negative control (naïve sample) needed to be below an OD of 0.24 for serum and 0.05 for BAL fluids and was defined as assay cutoff. Serum samples with an OD value below the assay cutoffs were negative and given the arbitrary value of 1.

RSV-specific IgA levels in BAL fluids were measured by a direct ELISA using a standard curve. 96-well ELISA plates were coated overnight with RSV antigen (Meridian). Samples were used at dilutions between 1:20 and 1:200 and detected with goat anti-mouse IgA-HRP (Invitrogen). The antibody titers were calculated by a 4-parameter fit (Magellan Software). The average of the OD values of the negative control (naïve sample) needed to be below an OD of 0.140, which was defined as assay cutoff. Serum samples with an OD value below the assay cutoff were negative and given the arbitrary value of 1.

For data presentation, the geometric mean of the antibody titers (GMT) with 95% confidence interval (CI) was calculated using GraphPad Prism version 8.4.3.

### Statistical Analysis

The analysis on antibody titers was done on the log10-transformed data. Antibody titers and viral load were analyzed using a normality test on the log10-transformed data (Shapiro–Wilk and an equal variance test [Brown–Forsythe] should the data pass normality). In case the normality test did pass, the ANOVA was followed by the Tukey all pairwise multiple comparison procedures test to compare the groups. If the normality did not pass, we used a non-parametric method (Kruskal–Wallis one-way analysis of variance on ranks) followed by a multiple comparison procedure (Dunn’s method). When only two groups were compared, a *t*-test was used in case of normality; otherwise, the Mann–Whitney rank sum test was used.

## Results

### Protection Against RSV Is Impaired in the Absence of CD4 or CD8 T Cells

**Figure 1:**
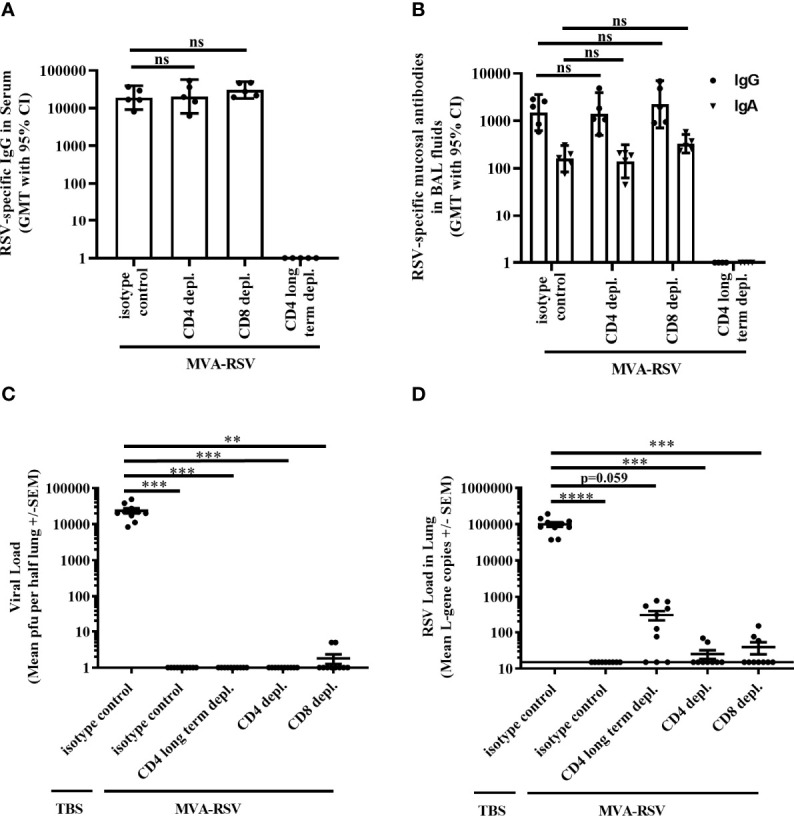
Effect of T-cell depletion on efficacy. BALB/c mice (*n* = 5) either were vaccinated (IN) twice 3 weeks apart with 1 × 10^8^ TCID_50_ MVA-RSV or received TBS. RSV challenge (IN, 10^6^ pfu) was performed 2 weeks after the last vaccination. One day prior and 1 day after RSV challenge, mice were either treated (IP) with an anti-CD4 (CD4 depl.) or anti-CD8 (CD8 depl.) or an isotype-matched control antibody (isotype control). For long-term depletion of CD4 T cells, injection with the anti-CD4 antibody was performed 2 days before prime vaccination and thereafter twice a week. **(A)** RSV-specific IgG in serum was measured on Day 33 (12 days post second vaccination) by ELISA. Titers were comparable between vaccinated mice treated with the isotype control antibody and anti-CD4 or anti-CD8 antibody (*p* = 0.9965 for CD4-depleted mice and *p* = 0.5426 for CD8-depleted mice; Tukey test). **(B)** RSV-specific mucosal IgA and IgG in BAL fluids were measured on Day 39 (4 days post challenge) by ELISA. Mice treated with anti-CD4 or anti-CD8 antibody had comparable RSV-specific mucosal IgA (*p* = 0.9717 for CD4-depleted mice and *p* = 0.1087 for CD8-depleted mice, Tukey test) as well as mucosal IgG (*p* = 0.9991 for CD4-depleted mice and *p* = 0.8205 for CD8-depleted mice, Tukey test) to vaccinated mice treated with the isotype control antibody. No antibodies could be measured in the CD4 long-term depleted group. For both panels the geometric mean titers (GMT) with 95% confidence interval (CI) are shown. Representative data of one out of two mouse studies are shown. ns: not statistically significant. Four days post challenge mice were sacrificed and the viral load in lung was measured by **(C)** plaque assay. Mean pfu per half lung ± SEM is shown. **(D)** Viral load detected by RT-qPCR. Mean L-gene copies ± SEM is shown; the black line indicates the lower limit of detection (LLOD = 15 gcs). Plaque assay data showed significant differences between TBS-treated control mice and all other groups (***p < 0.0005, **p < 0.005 Dunn’s method). RT-qPCR data showed significant differences between TBS-treated control mice and vaccinated control mice, CD4 depl. mice, and CD8 depl. mice (****p < 0.00005, ***p < 0.0005 Dunn’s method). Pooled data of two separately performed mouse studies are shown (*n* = 9 or 10).

To elucidate the role of T cells in RSV clearance from the lungs after challenge of vaccinated mice, we experimentally depleted CD4 or CD8 T cells, which is an established method to assess the role of T cells during immune responses, already used in various experimental systems (10, 17–20). For that purpose, four groups of BALB/c mice were vaccinated with MVA-RSV in a prime-boost regimen 3 weeks apart (**Supplementary Figure 1A**). One group (CD4 long-term depleted) was treated with a CD4-depleting antibody before vaccination and throughout the vaccination and challenge phase (**Supplementary Figure 1B**). During RSV infection, this group had no CD4 T cells in blood or spleen (**Supplementary Figure 2**) and no RSV-specific IgG antibodies in serum or RSV-specific IgA and IgG in bronchioalveolar (BAL) fluids (**Figures 1A, B**). The lack of IgG and IgA isotypes was expected due to the lack of T-cell help for antibody class switch during the vaccination phase. Two additional groups of MVA-RSV-vaccinated mice were either treated with an CD4- or CD8-depleting antibody 1 day before RSV challenge and during the challenge phase (**Supplementary Figure 1C**). As intended, mice in these groups lacked either CD4 or CD8 T cells with a depletion efficacy of >99% for both T-cell subsets (**Supplementary Figure 2**). In contrast to the CD4 long-term depleted group, these two groups had comparable antibody titers for RSV-specific serum IgG (GMT of 20,325 [95% CI, 7,241 to 57,049] for CD4-depleted mice and GMT of 30,251 [95% CI, 18,094 to 50,576] for CD8-depleted mice), mucosal IgG (GMT of 1,404 [95% CI, 501 to 3,935] for CD4-depleted mice and GMT of 2,243 [95% CI, 711 to 7,070] for CD8-depleted mice), and mucosal IgA (GMT of 141 [95% CI, 63 to 316] for CD4-depleted mice and GMT of 331 [95% CI, 212 to 517] for CD8-depleted mice) to mice treated with an isotype-matched control monoclonal antibody (GMT of 18,874 [CI, 9,085 to 39,210] for RSV-specific serum IgG, GMT of 1,495 [95% CI, 623 to 3,588] for RSV-specific mucosal IgG, and GMT of 160 [95% CI, 84 to 303] for RSV-specific mucosal IgA).

The effect of the respective T-cell-type depletion on protection against RSV infection was assessed 4 days post RSV challenge by measuring the presence of live virus in the lungs by plaque assay. Lungs from MVA-RSV-vaccinated, isotype-matched control antibody-treated mice that were shown to contain CD4 and CD8 T cells as well as RSV-specific IgG and IgA (**Supplementary Figure 2** and **Figures 1A, B**) were free from infectious virus (**Figure 1C**), while non-vaccinated (TBS-treated) mice given the isotype control antibody harbored high amounts of infectious RSV in their lungs (mean plaque-forming units [PFU]: 23,596). Interestingly, as judged by plaque assay 4 days post challenge, the lungs of CD4 long-term depleted and CD4-depleted vaccinated mice were also free of infectious RSV (**Figure 1C**), despite the absence of CD4 T cells and in case of CD4 long-term depletion, even in the absence of RSV-specific IgG and IgA (**Figures 1A, B**). In contrast, small amounts of infectious virus (5 PFU, each) were present in 2 out of 10 CD8-depleted mice (**Figure 1C**). These results suggested a role for CD8 T cells for complete clearance of infectious virus from the lung, but potentially no role for CD4 T cells or IgG and IgA. However, the plaque assay is not the most sensitive method, and a finer grading of efficacy can be achieved by using the more sensitive RT-qPCR method measuring RSV L-gene transcripts in the lung. In this assay, in line with expectations, lung samples from TBS-treated animals showed more than 4-fold higher viral loads (mean genomic copies [gcs] of 99,453) compared to the same samples by plaque assay, whereas samples from MVA-RSV-vaccinated mice treated with the isotype-matched control monoclonal antibody were still completely free of viral transcripts (**Figure 1D**). These results not only confirmed sterilizing mucosal immunity conferred by MVA-RSV in the presence of all arms of the immune system, but also indicated that the RT-qPCR assay offers a wider bandwidth to measure protection. Indeed, though detectable RSV L-gene transcripts in the lung were reduced by a factor of >2,500 for CD4- and CD8-depleted mice (*p* < 0.05) and a factor of 318 for CD4 long-term depleted mice compared to TBS-treated mice (**Figure 1D**), low amounts of viral transcripts were still detectable not only in CD8 T-cell-depleted mice (3 of 10 mice, mean gcs of 39) as anticipated based on the plaque assay results, but also in CD4 T-cell-depleted mice (2 of 9 mice, mean gcs of 25), indicating that CD4 as well as CD8 T cells play a role for complete viral clearance in the lung. This effect was even more pronounced in the CD4 long-term depletion group, which was lacking CD4 T cells already during the vaccination phase and thereby also IgG and IgA, due to the inability of antibody class switch in the absence of CD4 T-cell help. In this group, more than half of the mice (7 out of 10) had up to 786 gcs, compared to 2 out of 9 mice with up to 69 gcs in the group that were CD4 depleted later, i.e., at the time of challenge. This result suggests that besides T cells, IgG and/or IgA antibodies contribute to mediating sterilizing immunity.

### The Importance of T Cells and Antibodies for Complete RSV Clearance Is Confirmed in Knockout Mice

**Figure 2:**
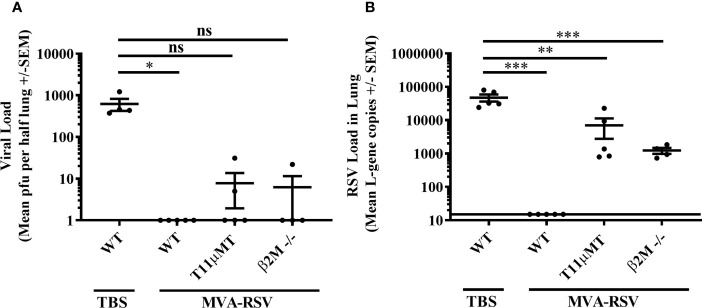
Efficacy in CD8 T cell (β2M -/-)- or antibody (T11µMT)-deficient C57BL/6 mice. C57BL/6 WT mice (*n* = 5), T11µMT (*n* = 5), or β2M -/- (*n* = 4) were vaccinated (IN) twice 3 weeks apart with 1 × 10^8^ TCID_50_ MVA-RSV. WT mice treated with TBS served as controls. RSV challenge (IN, 10^6^ pfu) was performed 2 weeks after last vaccination. Four days post challenge mice were sacrificed, and the viral load in lung was measured by **(A)** plaque assay. Mean pfu per half lung ± SEM is shown. **(B)** Viral load detected by RT-qPCR. Mean L-gene copies ± SEM is shown; the black line indicates the lower limit of detection (LLOD = 15 gcs). Plaque assay data (pfu) showed significant differences between TBS-treated control mice and vaccinated control mice (*p < 0.05, Dunn’s method). RT-qPCR data (L-gene copies) showed significant differences between TBS-treated control mice and all other groups (***p < 0.0005, **p < 0.005, Tukey test). ns: not statistically significant.

Next, we wanted to know whether these findings held true in a different, potentially even cleaner setting. For this reason, we used mice lacking CD8 T cells due to missing a constituent of major histocompatibility class I molecules (β2M -/-; 21, 22) or mice unable to produce RSV-specific antibodies due to a rearranged heavy chain gene specific for a VSV virus (T11µMT; 23), which are well-established models to assess the role of CD8 T cells or antibodies, respectively (24–26). Both have a C57BL/6 genetic background that is less sensitive to RSV infection (27–30). Similar to the BALB/c mouse experiments described above, C57BL/6 wild-type (WT) mice, β2M -/-, or T11µMT mice were vaccinated twice with 1 × 10^8^ TCID_50_ of MVA-RSV, challenged 2 weeks later with RSV, and the presence of RSV was assessed 4 days post challenge in the lungs by plaque assay and RT-qPCR (**Supplementary Figure 1A**). As expected, non-vaccinated C57BL/6 WT mice showed a lower viral load after challenge than BALB/c mice (621 versus 23,596 mean PFU by plaque assay and 47,300 versus 99,453 mean gcs by RT-PCR, **Figures 2** vs. **Figures 1C, D**). Complete absence of infectious virus as well as RSV specific L-gene transcripts in the lungs of MVA-RSV-vaccinated, RSV-challenged WT C57BL/6 mice (**Figures 2A, B**) confirmed that sterilizing mucosal immunity is also vaccine mediated in the C57BL/6 mouse model. Plaque assay results showed that in contrast to TBS control mice with high viral loads (mean PFU of 621), vaccinated β2M -/- mice were either able to fully clear infectious virus from their lungs or showed only small amounts (22 PFU) in one out of 4 mice (**Figure 2A**). All vaccinated β2M -/- mice had detectable RSV-specific transcripts (mean gcs of 1,224) determined by RT-qPCR (**Figure 2B**), confirming the importance of CD8 T cells for complete RSV clearance from the lungs seen in the CD8-depleted mice.

Since viral clearance from the lungs seemed to be more impaired in the absence of CD4 T cells and IgG and/or IgA antibodies as indicated by the results of the CD4 long-term depleted mice, the impact of antibodies for viral clearance was further investigated in T11µMT transgenic mice that also lack RSV-specific IgM. Here, infectious virus was detected in 2 out of 5 vaccinated T11µMT transgenic mice. The difference in RSV-specific viral transcripts between T11µMT transgenic mice (mean gcs of 7,005) and TBS control mice (mean gcs 47,300) was 7-fold (*p* < 0.005), accrediting RSV-specific antibodies a role in protection from RSV.

Although data from the transgenic and knockout mice are limited to a smaller number of animals, the obtained results are valuable in that they support the findings in the immunodepleted BALB/c mice in a second mouse model, reiterating that T cells in combination with antibodies are required for complete protection.

### RSV-Specific Serum Antibodies Alone Afford Only Weak Protection

**Figure 3:**
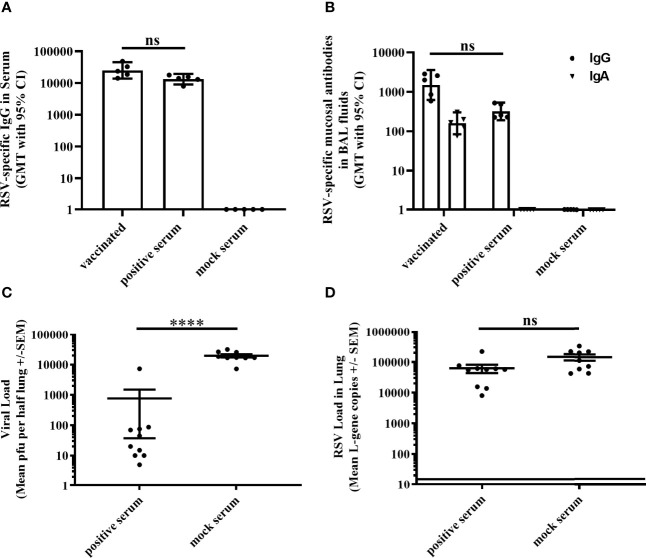
Efficacy of passively transferred RSV-specific antibodies. Either BALB/c mice (*n* = 5) were vaccinated (IN) twice 3 weeks apart with 1 × 10^8^ TCID_50_ MVA-RSV or non-vaccinated BALB/c mice received 1 ml of serum (IP) from MVA-RSV-vaccinated (positive serum) or mock-vaccinated control mice (mock serum) 1 day before challenge. **(A)** RSV-specific IgG in serum was measured on Day 39 (4 days post serum transfer) by ELISA. Geometric mean titers (GMT) with 95% confidence interval (CI) are shown. Titers were comparable between MVA-RSV-vaccinated mice and mice receiving positive serum (*p* = 0.4127, Dunn`s method). **(B)** RSV-specific mucosal IgA and IgG in BAL fluids were measured on Day 39 (4 days post serum transfer) by ELISA. GMT with 95% CI is shown. RSV-specific mucosal IgG titers were comparable between vaccinated mice and mice receiving RSV-specific serum antibodies (*p* = 0.2313, Dunn’s method). For **Figures 3B** and **1B**, identical results for RSV-specific mucosal IgA and IgG for MVA-RSV-vaccinated control animals were shown as this experiment was run in parallel to reduce the number of animals required. **(C)** Viral load in lung was measured 4 days post RSV challenge (IN, 10^6^ pfu) by plaque assay. Mean pfu per half lung ± SEM is shown. Viral load (pfu) determined by plaque assay was significantly (****p < 0.00005;Mann–Whitney *U*-test) reduced by the passive transfer of RSV-specific antibodies compared to the viral load of mice receiving the mock serum. **(D)** Viral load detected by RT-qPCR. Mean L-gene copies ± SEM is shown; the black line indicates the lower limit of detection (LLOD = 15 gcs). There was no statistically significant difference (*p* = 0.066, Mann–Whitney *U*-test) in RSV-specific L-gene copies between mice receiving RSV-specific antibodies and mice receiving the mock serum. Pooled data of two separately performed mouse studies are shown (*n* = 9 or 10). ns: not statistically significant.

Given the limited protection afforded in the absence of RSV-specific antibodies in T11µMT mice, we evaluated the sole effect of antibodies for protective efficacy. Serum from MVA-RSV-vaccinated mice (positive serum) was passively transferred to previously naïve BALB/c mice 1 day prior to RSV challenge (**Supplementary Figure 1D**). As a control, normal serum from naïve mice (mock serum) was transferred. The transfer of positive serum led to a detectable RSV-specific IgG antibody titer in serum (**Figure 3A**) and lung mucosa (**Figure 3B**) 4 days post challenge that was comparable in titers to vaccinated WT BALB/c mice (*p* = 0.4127 for IgG serum titers and *p* = 0.2313 for IgG BAL titers), while RSV-specific mucosal IgA could not be measured in the lungs of mice receiving positive serum. Four days after challenge with RSV, plaque assay results revealed that infectious RSV was 26-fold reduced (*p* < 0.00005) by the passive transfer of RSV-specific antibodies compared to the viral load of mice receiving mock serum, but still measurable in all mice (**Figure 3C**). RSV L-gene transcripts detected by RT-qPCR showed only a slight (factor of 2), not statistically significant (*p* = 0.066) difference between the positive and mock serum transfer groups (**Figure 3D**), indicating that RSV-specific serum antibodies and mucosal IgG alone could not eliminate RSV from the lung.

### Mucosal IgA Contributes to Complete Protection

**Figure 4:**
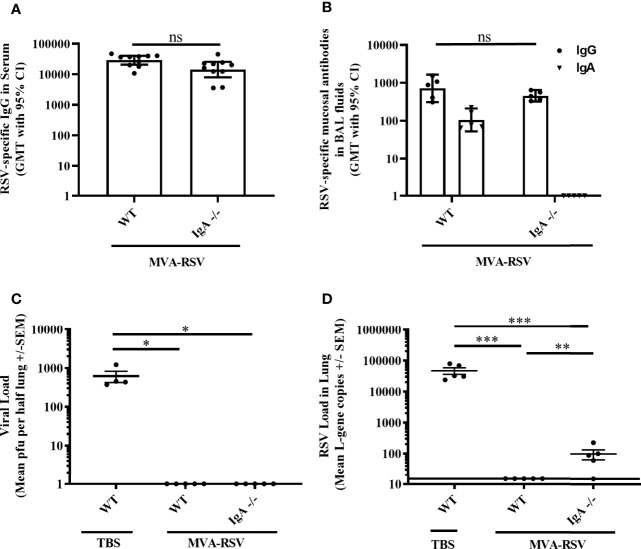
Role of IgA for RSV protection. C57BL/6 WT (*n* = 5 to 10) or IgA-deficient (IgA-/-, *n* = 5 to 10) mice were vaccinated (IN) twice 3 weeks apart with 1 × 10^8^ TCID_50_ MVA-RSV. C57BL/6 WT mice treated with TBS served as controls. **(A)** RSV-specific IgG in serum was measured on Day 34 (13 days post last vaccination) by ELISA. Geometric mean titers (GMT) with 95% confidence interval (CI) are shown. Titers were similar between vaccinated WT mice and IgA -/- mice (*p* = 0.054, Mann–Whitney *U*-test). **(B)** RSV-specific mucosal IgA and IgG in BAL fluids were measured on Day 39 (4 days post challenge) by ELISA. GMT with 95% CI is shown. Mucosal IgG titers were comparable between vaccinated WT mice and IgA -/- mice (*p* = 0.4206, Mann–Whitney *U*-test). Mucosal IgA was only detected in vaccinated WT mice but not in IgA -/- mice, as expected. **(C)** Viral load in lung was measured 4 days post RSV challenge (IN, 10^6^ pfu) by plaque assay. Mean pfu per half lung ± SEM is shown. The lungs of vaccinated IgA -/- mice were completely free from infectious virus analogous to the vaccinated WT control group (*p < 0.05; Dunn’s method). **(D)** Viral load detected by RT-qPCR. Mean L-gene copies ± SEM is shown; the black line indicates the lower limit of detection (LLOD = 15 gcs). L-gene copies were significantly reduced in IgA -/- mice compared to TBS-treated WT mice (**p < 0.005, ***p < 0.0005, Tukey test). ns: not statistically significant. For **Figures 4C, D** and **2A, B**, identical results for plaque assay (pfu) and RT-qPCR (L-gene copies) for TBS-treated and vaccine-treated control animals were shown as this experiment was run in parallel to reduce the number of animals required.

Knowing that RSV-specific IgG in serum and at mucosal sites did not achieve complete protection on its own, we looked at the role of mucosal IgA for RSV clearance in C57BL/6 mice lacking IgA (IgA -/-; 31, 32). At the time of RSV challenge, IgA -/- mice vaccinated with MVA-RSV had comparable levels of RSV-specific IgG in serum (*p* = 0.0539; **Figure 4A**) and lung mucosa (*p* = 0.4206; **Figure 4B**) to vaccinated WT C57BL/6 mice. Comparable RSV-specific T cells were also present, as supposed, since the development and function of CD4 and CD8 T cells are not impaired in IgA -/- mice (31). As expected, RSV-specific mucosal IgA was only measured in the vaccinated WT control mice and was absent in IgA -/- mice (**Figure 4B**). Four days after challenge, the lungs of vaccinated IgA -/- mice were free from infectious virus analogous to the vaccinated WT control group (**Figure 4C**) and only minimal viral load (mean gcs of 96) determined by RT-qPCR was detectable in the lungs of IgA -/- mice (**Figure 4D**) compared to TBS-treated WT mice (*p* < 0.0005). Comparing the viral transcripts detectable in IgA -/- mice and T11µMT transgenic mice (mean gcs of 96 versus 7,005), it appeared that the absence of IgA alone had a smaller impact on protection than the absence of all RSV-specific antibodies. Nevertheless, the fact that detectable RSV transcripts remained in vaccinated IgA -/- mice compared to vaccinated WT mice (*p* < 0.005) showed that complete protection relies on mucosal IgA as well.

## Discussion

Currently, immune mechanisms responsible for complete protection against RSV are still not fully identified. In mice, RSV-specific antibodies appeared to have only a minor role in the resolution of infection (33), and cellular immune responses are mainly thought to play an important role in clearing RSV, since RSV clearance from the lung could be mediated by an adoptive RSV-specific T-cell transfer in infected nu/nu mice (8) and duration of RSV shedding was shortened in the presence of RSV-specific CD4 and CD8 T cells (10). Similarly in humans, the highest level of neutralizing antibodies could not reliably protect against RSV re-infection, while on the other hand, individuals with low titers could be resistant (2, 34). Indeed, a vaccine candidate eliciting high neutralizing antibody responses has failed to show protective efficacy (35), suggesting that besides neutralizing antibodies, other arms of the immune system may also play an important role in protection against RSV disease. Human observational studies underline this notion, by highlighting the importance of T cells for viral clearance as well (11).

The MVA-RSV vaccine candidate used here was previously tested in a phase 1 and phase 2 clinical trial and shown to be safe and immunogenic. MVA-RSV induced both broad T-cell responses against all encoded RSV antigens and humoral responses against RSV A and RSV B (12, 13). These results suggest that MVA-RSV may activate various adaptive immune responses against RSV that could contribute to different pathways of protection. In fact, MVA-RSV was efficacious in a recent human challenge trial, demonstrating a vaccine efficacy of 79.3% in preventing moderately symptomatic RSV infection (manuscript in preparation).

To illuminate the immune mechanisms of protection against RSV upon MVA-RSV vaccination, we used mouse models in which a prime-boost vaccination of MVA-RSV provided sterilizing mucosal immunity 4 days after RSV challenge, indicated by the absence of infectious virus and the complete lack of RSV L-gene transcripts determined in the lung by plaque assay and RT-qPCR, respectively. *In vivo* depletion of T cells in mice has been extensively performed to study the role of T-cell subsets in various immune responses, such as antibody responses (36–38) and control of bacterial (39), viral (10, 40–42), fungal (43), or parasitic infections (44). Hence, to elucidate the role of the cellular immune responses for RSV protection, we depleted CD8 or CD4 T cells 1 day prior and during the challenge phase and, in the case of the CD4 T cells, additionally before and during vaccination and the challenge phase. Furthermore, CD8 T-cell-deficient β2m -/- mice were employed.

Data presented here demonstrate that protection afforded by MVA-RSV was only partial in the absence of CD4 or CD8 T cells. Both vaccinated CD8 T-cell-depleted mice (2 of 10) and vaccinated β2M -/- mice (1 of 4) could not completely clear infectious virus from their lungs as judged by plaque assay 4 days post RSV challenge. Moreover, RSV-specific transcripts were detected by RT-qPCR at small levels in all vaccinated β2M -/- mice and also in vaccinated CD8-depleted mice (3 of 10). Although differences in the genomic copy numbers between the CD8-depleted mice (BALB/c) and the β2M -/- mice (C57BL/6) were expected due to the different genetic background and RSV susceptibility of the mouse strains, it was apparent that upon CD8 depletion, only 3 of 10 mice showed low detectable genomic copy numbers compared to the β2M -/- mice, which all showed clearly detectable copies of the RSV L-gene. One explanation might be an incomplete CD8 T-cell depletion especially for RSV-specific vaccination-induced CD8 T cells at the mucosal respiratory areas in mice treated with anti-CD8 antibodies, which was not analyzed. However, both groups clearly indicated that CD8 T cells are important to mediate sterilizing mucosal immunity, and this finding is also consistent with CD8 T-cell depletion data in mice (4) or data from other animal models (e.g., calves), suggesting a role for CD8 T cells in limiting RSV replication (45).

An earlier study demonstrated that CD4 T cells, important for the induction of mucosal and systemic RSV-specific antibodies, were necessary for RSV clearance shown by adoptive CD4 T-cell transfer experiments into RSV-infected nude mice (46). Similar results were seen in our study in the BALB/c mice treated with the two different CD4 T-cell depletion regimens. While no infectious RSV could be detected by plaque assay in vaccinated CD4 short- or long-term depleted mice, low amounts of viral transcripts were detected by RT-qPCR in a few CD4 short-term depleted mice (2 of 9) and in more than half of the CD4 long-term depleted mice (7 of 10), implicating a role of CD4 T cells for mediating sterilizing mucosal immunity.

Mice depleted for CD4 after vaccination had 11 times lower genomic copies present in their lungs than the CD4 long-term depleted group, which were depleted already before the vaccination. CD4 T cells could also play a role for the induction and expansion of CD8 T cells. However, a major role for this is not strongly supported by our data since the CD4 long-term depletion showed, in the plaque assay, less effect than the depletion of CD8 T cells alone. As shown here, the CD4 long-term depleted mice did not mount RSV-specific IgG and IgA, since immunoglobulin isotype switching from IgM depends on the presence of CD4 T-cell help. This likely contributed to an even lower protective efficacy compared to mice lacking only CD4 T cells during the challenge. Based on these results, it was evident that antibodies also play a role for complete RSV clearance from the lungs, which was further investigated in T11µMT transgenic mice (C57BL/6). These mice were unable to produce RSV-specific antibodies upon vaccination but had normal levels of RSV-specific T cells at the time of RSV challenge. Plaque assay results revealed that 2 out of 5 vaccinated T11µMT mice could not completely clear infectious RSV from their lungs and viral transcripts were only slightly reduced compared to non-vaccinated WT mice, confirming that antibodies were required for complete protection. Similar results were observed in experiments with CD4 long-term depleted mice and B-cell-deficient mice (anti-µ treated), showing that RSV-specific antibody responses were important for complete protection from RSV replication in lungs (5).

However, passive transfer of RSV-specific antibodies to previously naïve BALB/c mice 1 day prior to RSV challenge had only a minor protective effect. We could show that mice receiving RSV-specific antibodies exhibit RSV-specific IgG in serum and mucosa (BAL fluids), but no mucosal IgA at the time of challenge, indicating that RSV-specific antibodies and mucosal IgG alone afford only weak protection against RSV. Though the induction of neutralizing antibodies has not explicitly been addressed in this study, earlier unpublished mouse studies showed that MVA-RSV vaccination induces neutralizing antibodies at comparable levels as RSV exposure. These findings raised the question on the role of mucosal IgA for RSV protection as it could act as a first-line immune defense against the virus (47). Several studies in infants and adults revealed the importance of the mucosal humoral response for protection against RSV. RSV-specific nasal IgA correlated more strongly with protection from PCR-confirmed infection than serum neutralizing antibodies (3), and higher nasal IgA predicted a lower infectivity and lower levels of RSV replication (48). Our data obtained in vaccinated and challenged IgA -/- mice showed that IgA is indeed required for complete protection, although IgA alone had a smaller effect on protection than the absence of all RSV-specific antibodies, indicated by the 73-fold higher genomic copies detected in the lungs of T11µMT transgenic mice compared to the IgA -/- mice.

In summary, after vaccination with MVA-RSV, clearance of RSV from murine lungs was only complete in the presence of RSV-specific antibodies, including mucosal IgA, as well as CD4 and CD8 T cells. These findings demonstrate that vaccination with MVA-RSV induces immune parameters from all arms of the adaptive immune system, which together warrant sterilizing protection against RSV exposure. Indeed, RSV-specific antibodies, including IgA, and T-cell responses against all RSV antigens encoded in MVA-RSV, were boosted in RSV-experienced human subjects (12, 13) and support the notion that an effective RSV vaccine needs to induce a balanced humoral and cellular immunity.

## Data Availability Statement

The original contributions presented in the study are included in the article/**Supplementary Material**. Further inquiries can be directed to the corresponding author.

## Ethics Statement

The animal study was reviewed and approved by the Government of Upper Bavaria.

## Author Contributions

KE: Supervision, Project administration, Investigation, and Writing review and editing. YW: Project administration. JH: Investigation. CB: Investigation. MF: Investigation. AH: Investigation. MS: Conceptualization and Methodology. MK: Vaccine design and production. HH: Conceptualization, Methodology, and Writing review and editing. PC: Conceptualization, Methodology, and Review. AV: Conceptualization, Methodology, Supervision, and Writing review and editing. All authors contributed to the article and approved the submitted version.

## Conflict of Interest

KE, JH, CB, MF, MK, HH, AV, and PC are employees of Bavarian Nordic. YW and AH were employed by Bavarian Nordic at the time of conducting this study. MS is employed as a consultant at Bavarian Nordic.

The authors declare that the study was funded by Bavarian Nordic. The funder was involved in the study design, collection, analysis, interpretation of data, the writing of this article, and the decision to submit it for publication.

## Publisher’s Note

All claims expressed in this article are solely those of the authors and do not necessarily represent those of their affiliated organizations, or those of the publisher, the editors and the reviewers. Any product that may be evaluated in this article, or claim that may be made by its manufacturer, is not guaranteed or endorsed by the publisher.
