# Supplementary material for: A Recombinant MVA-Based RSV Vaccine Induces T-Cell and Antibody Responses That Cooperate in the Protection Against RSV Infection
Source: Front Immunol. 2022 Jun 14;13:841471. doi: 10.3389/fimmu.2022.841471 (PMC9238321; doi:10.3389/fimmu.2022.841471)
Supplement: Supplementary file 1 [file DataSheet_1.pdf]

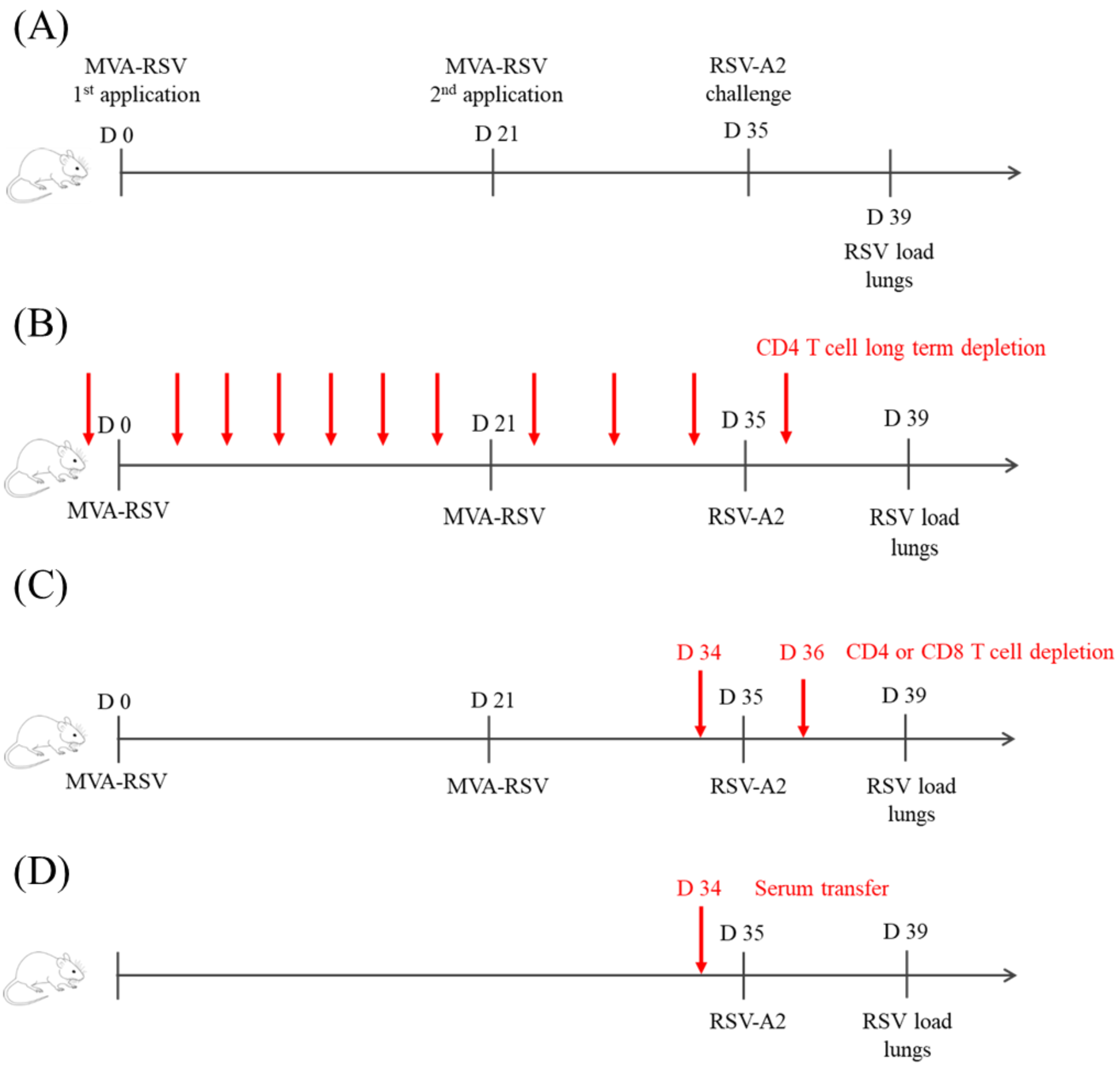

Supplementary Figure 1. Schematic representation of experimental designs. (A) Vaccination and challenge model in BALB/c and C57BL/6 mice: mice were vaccinated (IN) twice on Day 0 and 21 with 1x10^8 $TCID_{50}$ MVA-RSV. Mice treated with TBS served as controls. RSV challenge (IN, 10^6 pfu) was performed 2 weeks after last vaccination (Day 35). Four days post challenge (Day 39) mice were sacrificed and the viral load in lung was measured by plaque assay and RT-qPCR. Experimental depletion of CD4 or CD8 T cells: (B) for long term depletion of CD4 T cells, injection (IP) with an anti-CD4 antibody was performed 2 days before prime vaccination and thereafter twice a week. Viral load in lung was analyzed 4 days post RSV-A2 challenge (Day 39) (C) One day prior (Day 34) and one day after RSV challenge (Day 36) mice were either treated (IP) with an anti-CD4 or anti-CD8 antibody. As control an isotype matched control antibody was used. Viral load in lung was analyzed 4 days post RSV-A2 challenge (Day 39) (D) Serum transfer: non-vaccinated BALB/c mice received 1 mL serum (IP) from MVA-RSV vaccinated (positive serum) or mock vaccinated control mice (mock serum) one day before challenge (Day 34). Viral load in lung was analyzed 4 days post RSV-A2 challenge (Day 39). D: Day

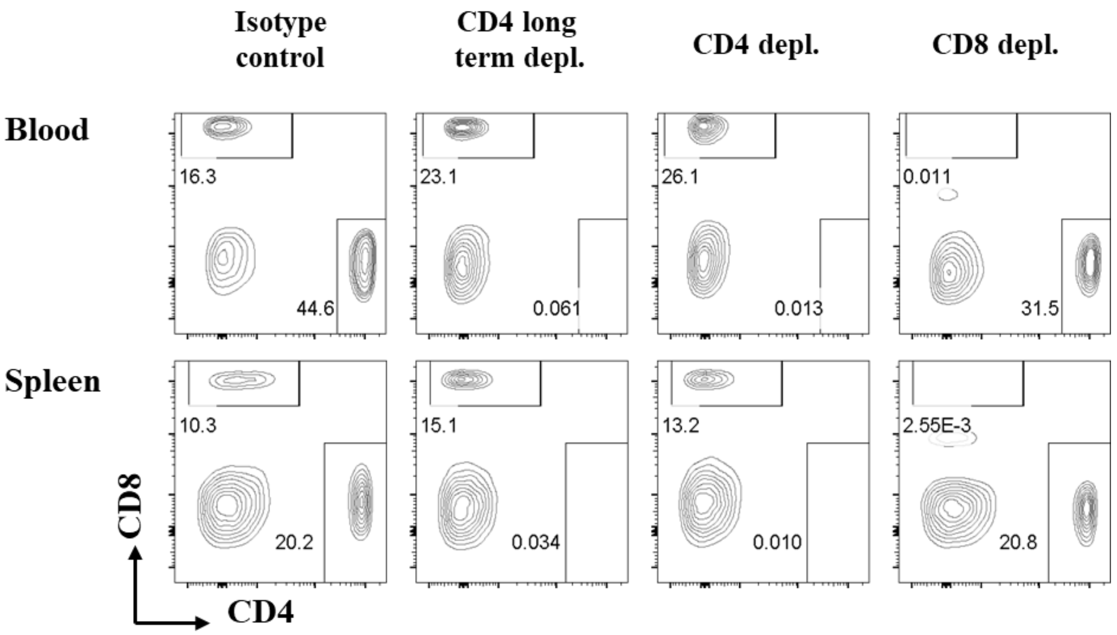

Supplementary Figure 2. Immune status of T cell depleted BALB/c mice. BALB/c mice (n=5) were either vaccinated (IN) twice 3 weeks apart with $1x10^8$ $TCID_{50}$ MVA-RSV or received TBS (control). RSV challenge (IN, 10^6 pfu) was performed 2 weeks after last vaccination. One day prior and one day after RSV challenge mice were either treated (IP) with an anti-CD4 (CD4 depl.) or anti-CD8 (CD8 depl.) or an isotype matched control antibody (isotype control). For long term depletion of CD4 T cells, injection with the anti-CD4 antibody was performed 2 days before prime vaccination and thereafter twice a week. Efficacy of these treatments was determined by FACS analysis of heparinized whole blood and splenocytes at Day 39 (4 days post RSV challenge).
